# A Persecuted Hospital

**Published:** 1892-03-12

**Authors:** 


### The Doctor's Armchair.

### A PERSECUTED HOSPITAL.

A Hospital can neither bark nor bite; and so it sometimes has rather a bad time of it at the hands of thoughtless and mischievous persons. A few years ago one of the lady officials of the London Hospital had the misfortune to mortally offend, though without intending it, certain other ladies, who had also once upon a time been associated with London hospitals. "A brother offended is harder to be won than a strong city," says an Eastern proverb. But a woman made deeply angry does not appear to know what the word "reconciliation" means. If the person who has made her angry have been a man, she may some time or other learn to forgive him, and even to take him to her jealous heart. But if the offender has, by an ill chance, been a sister woman, then nothing less will satisfy the outraged goddess than the shedding of the last drop of the offender's life-blood.

But it is difficult, and leads to inconveniences, to carry daggers and to shed life-blood in these days of a degenerate civilization. Men, the stronger sex, have tacitly agreed that revenge, which used to be considered "a feast for the gods," is altogether too expensive a dainty for them to indulge in. But a certain class of women, whose brains are in a much more protoplasmic and undifferentiated condition, cannot by any means bring themselves to this philosophic temper of mind. They still hate their enemies as the Jews of old used to do, and take a fierce pleasure in seeing them metaphorically dashed against the stones. A man, even in the pursuit of his revenge has some regard for innocent persons, and things that may happen to be accidentally or casually associated with his enemy. No Tory Member of Parliament, for example, would blow up the House of Commons merely because Mr. Labouchere is an occupant of its benches. Neither would a member of the Liberation Society set fire to the House of Lords because the Archbishop of Canterbury there sets forth his defence of the Church in Wales. But a woman, in the sublimity of her rage, is like Gallio, and cares for none of these things. The greater the catastrophe she can associate with her vengeance the greater and the more intense will her satisfaction be.

At the quarterly court of Governors of the London Hospital last week, the Chairman of the House Committee, Mr. Edward Murray Ind, declared that the income of the charity had fallen off by £10,000 during the year 1891. He said that, of course, the only method of dealing with such a deficit would be the closing of some of the wards. Now, why has the income fallen off? And what class of people is it who have withdrawn their gifts to this grand and altogether indispensable charity? If it were the permanent constituents of the hospital—that is, the regular annual subscribers—the fact would be one of the most painful gravity. But it is not. The annual subscribers, who have known all about the inner working of the institution for years, have been loyal to a man. There has been no falling off whatever in their contributions. It is the casual givers, and the casual givers alone, who have withheld from the largest, most economical, and most entirely indispensable hospital in London the sum of £10,000 during the past year. In other words, so far as they are concerned, they have made it necessary, unless an intelligent public will come to the rescue, to keep no less than a hundred of the beds at the London Hospital entirely vacant during the whole of the present year. But why have these casual supporters withheld their gifts? For no other reason but that certain ill-informed newspaper editors have chosen to insert in their papers unjust and misleading paragraphs about the hospital. And why have the newspaper editors inserted the misleading paragraphs? Because they are comparatively young, and have chosen to take their information at second-hand rather than to seek it at the fountain head; and because their informants have been willing to go all lengths, even though the final effect of their work should be the partial ruin of one of the noblest medical charities in the world.

Never were cause and effect more disproportionate. The whole thing would be entirely ridiculous if it were not for the gravity of the consequences. The London Hospital has not done a single thing which the most critical could stigmatise as really a fault. On the contrary, its managers have earned and secured the approval of all fair-minded and non-Pharisaical persons. At last week's meeting the Rev. J. F. Kitto, who is well-known, and was formerly an East-end vicar, spoke warmly of the good management of the House Committee, and moved a resolution expressing entire confidence in that committee, a resolution which, amid approving cheers, was carried unanimously.

What more can a body of Governors do than the governors of the London Hospital have now done? They have submitted all the charges made against the management of the hospital to the test of a comparison with the actual facts of the case. They have investigated the methods of the institution in every detail. They found, what was not surprising, that in certain particulars, alterations and improvements might be effected; and they made the alterations and improvements forthwith. But does anybody suppose that their action has satisfied the critics? Whoever is so foolish has studied human nature to little purpose. The critics, if we may judge by their conduct, are annoyed, rather than satisfied, that the improvements have been effected. The concessions which were yielded to their carpings have taken all the little wind out of their sails that they could ever boast. Improvement in the administration of the hospital was not the object of the real authors of the attack, who have kept steadily in the background. Their real aim was something very different, and something of which, if they had been animated by the smallest spark of rightmindedness, they would have been profoundly and lastingly ashamed.

But what is going to be done to make up the deficit of ten thousand pounds in the annual income of the hospital? Practical people must remember that it is the poor, and the poor alone, who are injured by the loss of this ten thousand pounds. The hospital must be kept open, and the salaries of the necessary officials paid, whether the income of the institution be great or small. No reasonable person can expect house Governors, Secretaries, Matrons, and nurses to work for nothing, any more than they can expect the premier or the indispensable policeman to work for nothing. We make a most urgent appeal to all our readers on behalf of the industrious poor of the East End of London, who will inevitably be deprived of the use of a hundred hospital beds for a whole year, unless intelligent and fair-minded persons will come to the rescue. The London Hospital has been wantonly persecuted; and though in the keen glare of adverse criticism it may have shown some microscopic deficiencies, yet its real grandeur and usefulness are as manifest to the eye as great mountains; whilst its honesty and splendid efficiency have never been questioned even by those who have been willing to damage it by repeated iteration of exploded charges. Those who have money to spare should give it quickly, both for the purpose of preventing the closing of several of the wards, and also as a protest against proceedings which threaten to cast a thousand sick and unoffending persons helpless into the street.

